# Prevalence and incidence of physical and sexual intimate partner violence perpetration among adolescent boys living with and without HIV

**DOI:** 10.4102/sajhivmed.v27i1.1780

**Published:** 2026-04-07

**Authors:** Phumla Madi, Candice W. Ramsammy, Avy Violari, Xiaoyue Zhang, Stefanie Hornschuh, Janan J. Dietrich, Busisiwe Nkala-Dlamini, Rachel Kidman

**Affiliations:** 1 Perinatal HIV Research Unit, Faculty of Health Sciences, University of the Witwatersrand, Johannesburg, South Africa; 2 Centre for Research in Health Systems, College of Health Sciences, University of KwaZulu-Natal, Durban, South Africa; 3 Biostatistical Consulting Core, School of Medicine, State University of New York at Stonybrook, Stony Brook, United States of America; 4 Health Systems Research Unit, South African Medical Research Council, Cape Town, South Africa; 5 Department of Social Work, Faculty of Humanities, University of the Witwatersrand, Johannesburg, South Africa; 6 Program in Public Health, State University of New York at Stony Brook, Stony Brook, New York, United States of America; 7 Department of Family, Population and Preventive Medicine, State University of New York at Stony Brook, Stony Brook, New York, United States of America

**Keywords:** HIV/AIDS, adolescents, emotional, sexual, physical intimate partner violence

## Abstract

**Background:**

Intimate partner violence (IPV) and HIV incidence rise substantially during adolescence. While surveys from African countries suggest that adult men living with HIV were more likely to be perpetrators of IPV, less is known about boys living with perinatal HIV (PHIV).

**Objectives:**

To measure the prevalence and incidence of IPV perpetration between adolescents who acquired HIV perinatally and their HIV-negative peers.

**Method:**

Adolescents 15–19 years old, in a sexual or dating relationship, were followed up for 1 year in Soweto, South Africa. We examined prevalence of IPV perpetration using a baseline survey and incidence using weekly prospective mobile phone surveys. Data were collected on emotional, physical, and sexual IPV. Associations between HIV status and IPV were estimated using logistic regression for lifetime and past-year perpetration, and generalised linear mixed models for past 24-h perpetration.

**Results:**

Of 498 participants, 251 were living with PHIV, and 247 were HIV-negative at baseline. Lifetime and past-year IPV perpetration were reported by 52% (PHIV 50%, HIV negative 53%) and 34% (PHIV 31%, HIV negative 37%), respectively. Over 12 000 weekly mobile surveys were submitted during follow-up. Participants reported 134 incidences of physical or sexual IPV out of 5032 occasions where the mobile survey coincided with participants seeing a partner. IPV incidence was estimated as three out of every hundred person-days. There was no significant difference in the incidence of perpetration by HIV status.

**Conclusion:**

In 3 out of 100 encounters with a partner, physical or sexual IPV perpetration was reported. Boys living with HIV were equally likely to perpetrate violence as HIV-negative boys. Interventions targeted at boys to reduce IPV behaviour should include all adolescents. In boys living with HIV who have regular contact with healthcare providers, there is an opportunity for an intervention to potentially reduce onward HIV transmission

**What this study adds:** Despite substantial research on IPV, little information exists on perpetration among adolescent boys who acquired PHIV. This study found similar incidence of IPV among participants with and without HIV from the same community.

## Introduction

Intimate partner violence (IPV) is defined as behaviours by a current or former intimate partner that cause emotional, physical, or sexual harm.^1,2^ During adolescence, both IPV and HIV incidence rise dramatically.^3,4,5^ Moreover, the relationship between these risks is bidirectional. Youth living with HIV may be victims as well as perpetrators of IPV, with some studies suggesting that adults living with HIV are more likely to experience IPV, resulting in part from relationship conflict as a result of HIV disclosure.^6^

In adults, a review of national cross-sectional surveys in 27 African countries found that adult men living with HIV were more likely to be perpetrators of IPV (adjusted prevalence ratio = 1.09; 95%CI: 1.01–1.16).^7^ There is also evidence, for example, that victims of IPV are up to twice as likely to contract HIV.^8^ In the adult population, the link between HIV and IPV can be explained by common risk factors such as poverty, adverse childhood experiences, substance use, and gender inequality views.^9,10,11^

While we do not know if this holds true for adolescent boys who have grown up with HIV and have a very different route of infection, some of the risk factors may be shared with their adult counterparts. In addition, boys living with HIV, particularly in impoverished contexts, may experience a higher burden of childhood adversity (e.g. orphanhood, witnessing domestic violence, etc.),^12,13^ which is a strong predictor of IPV perpetration^14,15^ in comparison to boys without HIV.

Adolescents of either gender can be perpetrators and victims of IPV, but research indicates that boys engage in more violent forms of IPV perpetration.^16^ Moreover, IPV behaviours start early. For example, a South African study showed that among Grade 8 boys, as young as 12 years of age, 46% reported emotional IPV perpetration and 39% reported physical or sexual IPV perpetration.^17^ Past studies highlight several reasons adolescent boys may perpetrate violence, including provocation by a partner, self-defence, stress and anger, as well as patriarchal cultures and dating norms.^18,19^

Understanding patterns of IPV perpetration among adolescent boys living with HIV, at an age when they begin to engage in sexual and romantic relationships,^20^ is critical for designing interventions and for preventing HIV transmission to their sexual partners.^21^ Moreover, behaviours are still modifiable during this developmental period; thus, there is an opportunity to intervene. In this study, we estimated the prevalence of IPV perpetration among adolescent boys (sex assigned at birth) and tested whether boys living with perinatally HIV (PHIV) would be more likely to perpetrate IPV as compared to their HIV-uninfected peers. In addition to examining lifetime and past-year prevalence through traditional retrospective surveys, we advance the field by prospectively capturing incidence. To do so, we used ecological momentary assessments that captured events in real time.^22^ A key strength of this approach is that it reduces recall bias and more accurately detects IPV perpetration.^23^ Moreover, it captures events as they occur in the boys’ natural environment (leading to better ecological validity), and better documents the key dynamics, such as motivation, surrounding each IPV event.^24,25^ Using the mobile phone data, we estimate the incidence of perpetration among adolescent boys in South Africa, describe who they victimise, and document the reported reasons for perpetration.

Based on the literature on childhood adversity and data from adults living with HIV, we hypothesised that adolescent boys with PHIV would perpetrate more IPV compared to their HIV-negative peers, both over their lifetime and within the past year.^26^ This study examines the prevalence and incidence of IPV among adolescent boys living with HIV and compares that to their HIV-negative peers.

## Research methods and design

This was a longitudinal cohort study in adolescent boys living with and without HIV.

### Study participants

Recruitment took place between November 2020 and May 2022 and covered 34 areas in Soweto to include all socioeconomic brackets. The planned study follow-up for each participant was 1 year. The PHIV cohort was recruited from public healthcare clinics; HIV-negative peers were recruited through community outreach in the same geographic areas. Their HIV-negative status was confirmed using a rapid HIV test. Over 1000 boys were approached, and boys who met the inclusion criteria were enrolled (251 PHIV and 247 HIV negative). Eligible participants had to be 15–19 years old, reside in and around Soweto, a peri-urban settlement in South Africa, and be either in a face-to-face dating relationship or had sex in the past month prior to enrolment. In addition, the PHIV cohort had to be aware of their HIV diagnosis and had to have a history of HIV infection before age 10, suggesting they were perinatally infected. Those with cognitive impairment that limited their ability to answer the survey were excluded from the study.

### Data collection and instruments

We used two methods for data collection: a survey questionnaire at baseline, taking place at the research site, followed by weekly ecological momentary assessments using a mobile phone over a period of 1 year. The baseline questionnaires were available in the local languages: English, IsiZulu, and Sesotho. To maintain confidentiality and remove desirability bias, the participants completed the baseline survey questionnaires on a tablet and submitted it on their own. A trained staff member was available on site to provide them with support as needed.

For the longitudinal mobile survey component, participants were provided with a mobile phone and completed an interactive training session at the baseline visit. They were assisted in downloading the SurveyCTO application (an online survey platform) onto the study mobile phone and were trained on how to use it. Further information on the mobile phone survey design and implementation is described elsewhere.^22^ Briefly, after the baseline visit and over a period of 52 weeks, participants received weekly requests to complete the mobile phone survey within 24 h. Each participant was assigned to receive the survey either on a weekday or on a weekend day. After 6 months, each participant was switched from receiving the survey on a weekday to receiving the survey on a weekend day or vice versa. Participants were provided with mobile data, enabling them to complete the survey, as well as a data incentive once they submitted the survey. The mobile phone survey data were managed by Ikapadata, a local South African digital health platform.

*Sociodemographic data* on age, race, schooling, relationship status, sexual activity, and age of sexual debut were collected in the baseline survey. As an indication of high-risk sexual behaviour, all participants were tested for a sexually transmitted infection (STI). Urine was tested for Chlamydia and Gonorrhoea using a multiplex PCR test (Cepheid Xpert CT/NG Assay, performed on the GeneXpert) for rapid detection and differentiation of genomic DNA from *Chlamydia trachomatis* (CT) and *Neisseria gonorrhoeae* (NG). Nucleic acid detection methods were also used to detect *Mycoplasma genitalium* and *Trichomonas vaginalis*.

*Lifetime and past-year IPV perpetration* were both measured in the baseline survey. We modified the WHO’s Violence Against Women Instrument^27^ to capture emotional and physical IPV perpetration in a gender-neutral manner with the help of the Adolescent Community Advisory Board. The scale captured both whether an event had ever happened, and whether it happened in the past 12 months. This tool has been used across other African populations^17^ and had good internal consistency when measuring perpetration (Cronbach’s α = 0.73–0.94).^27,28^ The Sexual Experiences Survey – Short Form Perpetration was used to measure acts of sexual violence perpetration.^29^ Items pertaining to intimate partners were adapted into five questions measuring lifetime and past-year perpetration. This tool has been used among a South African adolescent population and showed good internal reliability (Cronbach’s α = 0.80).^30^ Using the above, separate indicators were created for physical, emotional, and sexual perpetration; we also created a composite of any IPV perpetration.

*Past 24-h IPV perpetration* was measured in the mobile phone survey. If a participant indicated that they spent time with their girlfriend, boyfriend or other sexual partner in the past 24-h, they were prompted to answer a question on physical IPV perpetration (‘*In the past 24-hours, did you slap, shove, hit, kick or otherwise physically hurt your partner?*’). An additional question asked, ‘*When you have hurt or threatened to hurt your partner, what was the reason?*’ Reasons were generated based on a review of the literature on motivations for IPV perpetration,^31^ and participants were able to select multiple options. If the participant indicated that they had engaged in sexual activity during the past 24 h, they were asked who they had sex with and were asked a question on sexual IPV perpetration (‘*Did you use violence or coercion/threats to get them to have sex*?’).

### Statistical analysis

Chi-squared tests with exact *P*-values based on Monte Carlo simulation were utilised to compare the distribution of participants’ characteristics between PHIV and HIV-negative peers, including the proportion of participants who perpetrated IPV.

For lifetime and past-year IPV perpetration (any, emotional, physical, sexual) captured in the baseline survey, logistic regression models, with adjustment for age, race, and school enrolment, were constructed to analyse the association between HIV status and perpetration prevalence.

For past 24-h perpetration (any, physical, sexual) captured in weekly mobile phone surveys, generalised linear mixed-effect models considering participants as random effect were constructed to analyse the association between HIV status and perpetration incidence. Models adjusted for age, race, school enrolment, and mobile phone survey week.

Statistical analysis was performed using SAS 9.4 (SAS Institute, Inc., Cary, North Carolina, United States). LOGISTIC procedure was used to construct logistic regression models, and GLIMMIX procedure for generalised linear mixed effect models.^32,33^ Significance level was set at 0.05.

### Ethical considerations

The study protocol was approved by the Ethics Committee of the University of the Witwatersrand (reference number: 191001) and the Ethics Committee of Stony Brook University (reference number: IRB2019-00567), as well as by the local Department of Health who granted access to local HIV care clinics for recruitment. Participants were aware that participation in the study was voluntary and written informed consent and/or assent was obtained from adolescents and their caregivers. All adolescents were debriefed by a counsellor following the baseline survey, given a list of supportive resources, and provided a 24-h emergency number. Counselling support was also made available on the weekly mobile phone surveys through an easy-to-use request button at the end of each survey. This alerted a study counsellor, who would contact participants and help manage distress. Participants were referred to a psychologist or psychiatrist for further services when needed.

To maintain confidentiality of the data participants completed the electronic survey using only a study ID, and the data were uploaded to a secure server. The biostatistician had access to the survey data but not to the participants’ identity. For the mobile surveys, participants used a unique study ID and submitted to a secure, encrypted server. No information was stored on the phone once the survey was submitted.

## Results

At baseline, the majority of the 498 participants (71%, *n* = 352) were younger than 18 years and were attending school. Consistent with the enrolment criteria of either being in a face-to-face relationship or having had sex in the month prior to enrolment, nearly all were dating (99%, *n* = 493), and more than half were sexually active (52%, *n* = 255) (Table 1). Twenty participants (4%) had a positive urine test for one or more STIs, of whom only 15 reported being sexually active. There were nearly four times as many HIV-negative adolescents with an STI than PHIV. Overall, *C. trachomatis* was found in 14 participants, *N. gonorrhoeae* in six, *T. vaginalis* in two, and *M. genitalium* in one. Inconsistent condom use among those who were sexually active was also more prevalent in HIV-negative adolescents (Table 1).

**TABLE 1:** Baseline characteristics.

Characteristics		Total (*N* = 498)		PHIV (*n* = 251)		HIV negative (*n* = 247)		*P*-value†
		*n*	%	*n*	%	*n*	%	
Age (years)	15–17	352	71	177	71	175	71	0.935
	≥ 18	146	29	74	29	72	29	
Race	Black African people	458	92	227	90	231	94	0.206
	Other	40	8	24	10	16	6	
Enrolled in school	Yes	355	71	192	76	163	66	0.010
Have a girlfriend/boyfriend	Yes	493	99	249	99	244	99	0.692
Sexually active	Yes	255	52	94	38	161	66	< 0.001
Any sexually transmitted infection	Yes	20	4	4	2	16	7	0.006
Inconsistent condom use‡	Yes	79	34	16	20	63	41	0.001

† , For categorical variables, *P*-values were from Chi-square tests with exact *P*-values based on Monte Carlo simulation; ‡, Out of those who were sexually active who answered the questions on condom use: Total (*N* = 235), PHIV (*n* = 81), and HIV negative (*n* = 154).

### Prevalence of IPV

The prevalence of IPV perpetration is presented in Table 2. In the baseline survey, about half of the sample (52%, *n* = 258) reported perpetrating at least one form of IPV in their lifetime, and 34% (*n* = 171) reported doing so within the past year. Emotional IPV was the most frequently reported type of perpetration (39%, *n* = 196 lifetime; 26%, *n* = 129 past year); followed by sexual (27%, *n* = 135 lifetime; 13%, *n* = 64 past year), and physical IPV (17%, *n* = 85 lifetime; 10%, *n* = 51 past year).

**TABLE 2:** Prevalence and incidence of IPV perpetration by HIV status.

Variable		Total		PHIV		HIV negative	
		*n*	%	*n*	%	*n*	%
**Baseline survey perpetration**		*N* = 498		*n* = 251		*n* = 247	
Lifetime IPV	Any	258	52	126	50	132	53
	Emotional	196	39	90	36	106	43
	Physical	85	17	36	14	49	20
	Sexual	135	27	73	29	62	25
Past-year IPV	Any	171	34	79	31	92	37
	Emotional	129	26	55	22	74	30
	Physical	51	10	20	8	31	13
	Sexual	64	13	28	11	36	15
**Mobile phone survey perpetration (participant-level)†**		***N* = 420**		***n* = 214**		***n* = 206**	
Past 24-h IPV	Any	62	15	31	14	31	15
	Physical	54	13	26	12	28	14
	Sexual	23	5	11	5	12	6
**Mobile phone survey perpetration (survey-level observations)†,‡**		***N* = 5032**		***n* = 2535**		***n* = 2497**	
Past 24-h IPV	Any	134	3	64	3	70	3
	Physical	89	2	44	2	45	2
	Sexual	70	1	28	1	42	2

† , Numbers represent the number of participants who submitted a valid survey; IPV perpetration from mobile survey was collected only if a participant noted meeting with partner(s) in the past 24 h; data were collected on physical and sexual IPV only;

‡ , *n* values indicate number of observations.

### Daily incidence rate reported from mobile surveys

Compliance with submitting the mobile survey within 24 h of being prompted varied greatly (mean 49%, standard deviation 30%). We focus here on the 466 participants who submitted at least one valid mobile survey. These participants completed an average of 27 mobile phone surveys over the observation year. Over 12 000 valid mobile phone surveys were received (from 466 participants), of which 5032 were submitted on days where the participant spent time with a partner (from 420 participants). There were 134 reports of physical or sexual IPV perpetration: this amounts to IPV perpetration in 3% of encounters with a partner (Table 2). Among the 62 participants who reported physical or sexual IPV perpetration, 43 (69%) reported a single incident, while the remaining 19 (31%) reported multiple incidents, contributing to 91 events. Two per cent of observations (O) were physical (O = 89 of 5006) IPV, and 1% (O = 70, of 4940) sexual IPV perpetration. Expressed as an incidence rate, this translates into three cases in 100 person days. As shown in Table 2, there was no observed difference in the rates of past 24-h IPV by HIV status, including no difference for either physical or sexual perpetration.

### Differences in IPV perpetration by HIV status

The odds of any type of IPV perpetration in youth living with HIV were not significantly higher compared to youth without HIV, controlling for age, race, school enrolment, and survey week (Table 3). The finding was consistent when reporting lifetime, past year, or past 24 h IPV. Past-year emotional IPV perpetration demonstrated a trend, though non-significant, towards lower incidence among adolescents living with HIV compared to HIV-negative peers (OR = 0.70; 95% CI: 0.46–1.05; *P* = 0.0865).

**TABLE 3:** Estimated odds ratio and 95% confidence interval of IPV perpetration by HIV status based on multivariable logistic regression models (for baseline data) and GLMM models (for mobile ecological momentary assessments data).

Variables	Lifetime†			Past year†			Past 24 h‡		
	OR	95%CI	*P*-value	OR	95%CI	*P*-value	OR	95%CI	*P*-value
**Any IPV perpetration**									
PHIV	0.93	0.65–1.33	0.6800	0.80	0.55–1.17	0.2466	1.09	0.59–2.01	0.7823
Age: 15–17 years old	1.01	0.68–1.51	0.9468	1.12	0.73–1.70	0.6111	1.33	0.66–2.71	0.4268
Race: Black African people	1.58	0.81–3.06	0.1785	1.82	0.84–3.93	0.1292	1.70	0.46–6.27	0.4233
Enrolled in school	0.67	0.44–1.00	0.0526	0.84	0.55–1.27	0.4040	0.84	0.43–1.66	0.6233
**Emotional IPV perpetration**									
PHIV	0.79	0.55–1.14	0.2004	0.70	0.46–1.05	0.0865	-	-	-
Age: 15–17 years old	1.06	0.71–1.60	0.7721	0.94	0.60–1.48	0.7886	-	-	-
Race: Black African people	1.93	0.92–4.07	0.0828	2.37	0.90–6.22	0.0800	-	-	-
Enrolled in school	0.73	0.48–1.10	0.1271	0.70	0.45–1.10	0.1187	-	-	-
**Physical IPV perpetration**									
PHIV	0.72	0.45–1.17	0.1875	0.63	0.35–1.15	0.1314	1.08	0.58–1.98	0.8124
Age: 15–17 years old	1.15	0.67–1.95	0.6155	1.35	0.68–2.68	0.3883	1.42	0.69–2.90	0.3414
Race: Black African people	1.05	0.42–2.63	0.9112	1.35	0.40–4.61	0.6274	1.36	0.39–4.75	0.6260
Enrolled in school	0.54	0.33–0.90	0.0176*	0.71	0.38–1.35	0.2989	0.86	0.44–1.70	0.6721
**Sexual IPV perpetration**									
PHIV	1.24	0.83–1.85	0.2923	0.73	0.43–1.25	0.2533	0.96	0.36–2.53	0.9324
Age: 15–17 years old	1.12	0.71–1.76	0.6150	1.06	0.58–1.93	0.8529	1.58	0.49–5.14	0.4442
Race: Black African people	0.97	0.46–2.01	0.9257	1.30	0.44–3.82	0.6326	1.72	0.19–15.86	0.6329
Enrolled in school	0.84	0.54–1.32	0.4596	1.08	0.59–1.97	0.8133	0.73	0.25–2.10	0.5586

* , Significant difference *P* < 0.05.

† , *P*-values were from Type III tests based on multivariable logistic regression models with adjustment of age, race, and school enrolment;

‡ , *P*-values were from Type III tests based on generalised linear mixed effect model with adjustment of age, race, school enrolment, and mobile survey week.

### Reported reasons for physical and sexual IPV

Thirteen per cent of participants (*n* = 54 of 420) reported physical IPV during the mobile surveys, for a total of 89 events. The most frequently reported reason for hurting their partner was that they lost their temper (O = 27; 30%), followed by protecting themselves (O = 13; 15%), or to get attention or respect (O = 10; 11%). Other reasons are listed in Table 4.

**TABLE 4:** Reasons adolescent boys physically hurt their partner based on ecological momentary assessment.

Reasons for physical IPV perpetration	*n*	%
Lost temper or couldn’t control themselves	32	36
Self-defence or protection	13	15
Get their attention or make them listen or get respect	10	11
Drunk or high	9	10
Stressed or frustrated	4	5
Infidelity	4	5
Punishment	3	3
Upset about money	3	3
Refused to give a reason	11	12

Note: Reasons are presented across 89 mobile surveys containing reports of physical IPV perpetration; *n* values indicate number of observations.

Five per cent of participants (*n* = 23 of 420) reported sexual IPV during the mobile surveys, for a total of 70 events. On average, perpetrators reported that they used violence or coercion or threats in 31% of their sexual encounters (data not shown). A partner (girlfriend, wife, boyfriend, or husband was the victim in the majority (97%) of the sexual IPV perpetration events (Table 5).

**TABLE 5:** Reported victim of sexual IPV perpetration (from the mobile phone data).

Relationship to sexual partner	*n*	%
Girlfriend or wife	64	91
Boyfriend or husband	4	6
Female casual partner	3	4
Male casual partner	3	4
Female stranger	3	4
Male stranger	0	0

Note: These reflect the 70 mobile phone surveys containing reports of sexual IPV; multiple responses could be selected.

## Discussion

Over half of the adolescent boys surveyed in our study reported perpetrating IPV at some point their lifetime, with no difference based on HIV status. Contributing to the robustness of our findings, the study employed real-time data collection to report on the daily incidence rate of perpetration. We found that there were three events of IPV perpetration out of every 100 person days in which boys spent time with a sexual or romantic partner, highlighting the pervasive nature of the issue.

We observed few discernible differences by HIV status – a finding that contrasts with a review on adult men.^7^ The association in adults is attributed to similar vulnerabilities for both HIV and IPV.^18,34^ However, this relationship may not be present in our sample because of differences in mode of infection and its associated behavioural health risk factors.^35^ Specifically, those who acquire HIV behaviourally, as opposed to perinatally, may have pre-existing factors that lead them to engage in both IPV and risky sexual behaviours. Notably, excessive substance use, a significant risk factor, was more likely to be reported by adolescents who acquired HIV through behavioural choices.^35,36^ Our participants acquired HIV perinatally, and thus their risk pattern may differ.

In our study, IPV perpetration was common among both boys living with HIV and their peers. IPV perpetration has been shown to heighten the risk of HIV transmission.^37^ Therefore, it is crucial to ensure PHIV individuals are included in IPV prevention programmes. To develop responsive interventions, a comprehensive understanding of the context and drivers of IPV is imperative. Frameworks such as feminist theory, that consider gender and power dynamics, propose that IPV emerges as a manifestation of patriarchal structures and beliefs, which perpetuates male displays of power, control, and desires to dominate women.^18^ We anticipated that boys would endorse reasons for IPV perpetration that aligned with this framework, such as seeking respect and suspicion of infidelity. Indeed, seeking respect or attention was one of the common reasons for physical IPV. However, contrary to our expectations, the inability to control their temper and self- defence were the most frequently reported reasons. Adolescence is marked by hormonal fluctuations, academic pressure, and navigation of social relationships that often trigger strong emotions. Regulating these emotions and learning to control one’s temper is a developmental task that is being refined during this period and could be a target for intervention. We also found that sexual IPV was primarily directed at steady partners. Interestingly, some physical IPV may have been bidirectional, indicated by instances in which participants reported using violence in self-defence.

To our knowledge, this was the first prospective study using ecological momentary assessments to measure incidence of IPV perpetration amongst boys living with PHIV and compare it to a group of boys without HIV. There are, however, limitations to our study. Our sample had been engaged in long-term care, and may have been exposed to health information, attended HIV support groups, and had time to address stigma and other challenges with their trusted healthcare providers. Thus, they may not be representative of all boys living with HIV.^35^ Despite approaching PHIV in healthcare facilities, verifying each participant’s HIV status at birth would make the study unfeasible. That children known to be living with HIV before age 10 were perinatally infected is a very reasonable assumption in an area of high HIV prevalence among pregnant women, but the sample could have included boys with a different mode of transmission. Another limitation is the use of self-report measures, which could introduce recall and response bias.^38^ In addition, once-weekly surveys would likely bias reports of perpetration downward, making our estimates conservative. Also, to keep the surveys short, emotional IPV was not surveyed during the weekly mobile surveys.

A main strength of this study is that it complemented prevalence estimates from traditional surveys with estimates of daily incidence rates generated from daily diary methods. This approach reduced recall bias, and better captured IPV perpetration occurring in the boys’ natural environment. While the use of daily mobile phone surveys has several key advantages, there was substantial attrition. Compliance is a common obstacle in daily diary studies of IPV,^23,39^ and our focus on adolescents in a low-resource environment presented additional unique challenges.^22^ If participants were less likely to complete a mobile phone survey on days when they also perpetrated IPV, our results would again underestimate the true incidence.

There aren’t many violence interventions for adolescent boys. This is a key population to include in intervention programmes. As PHIV adolescents exhibit similar rates of physical and sexual IPV perpetration to their HIV-negative peers, tailored prevention interventions based solely on HIV status may not be a necessity. However, further research is essential to ascertain whether the underlying risk factors driving perpetration in both groups are, in fact, similar. Nevertheless, inclusive interventions are needed for PHIV adolescents, considering the heightened risk of HIV transmission associated with perpetration. Potential entry points for intervention include HIV support groups or other HIV care services. Intervention research may need to target relational and behavioural factors. This encompasses fostering healthy relationship norms, imparting conflict resolution skills, shaping attitudes towards IPV, and emphasising power negotiation within relationships – themes common to both groups of adolescent boys.^40,41^ These may be achieved through school programmes, embedding within existing local programmes for adolescent girls and young women, parenting life skills initiatives, and youth-friendly services at their local care clinics. Recognising the importance of early intervention, local care clinics need to identify at-risk individuals, facilitating timely and targeted efforts to address IPV and its associated risks among adolescents living with HIV. These findings are important because existing literature suggests that there are also negative internalised and externalised consequences for perpetrators of IPV, not just the victims.^42^

## Conclusion

HIV and IPV incidence increase rapidly during adolescence; however, few studies have looked at this behaviour among PHIV individuals. Bridging this gap in the literature, our study revealed alarmingly high rates of IPV perpetration, with no discernible differences by HIV status. This underscores the urgency for targeted interventions in all adolescents, ensuring that effective strategies encompass both prevention and support measures.
